# 2308 Community health workers as research advocates

**DOI:** 10.1017/cts.2018.242

**Published:** 2018-11-21

**Authors:** Amparo Castillo, Emily Anderson, Alicia Matthews, Raymond A. Ruiz, Wendy Choure, Kevin Rak, Marilyn Willis

**Affiliations:** Chicago Center for Clinical and Translational Science, University of Illinois

## Abstract

OBJECTIVES/SPECIFIC AIMS: Background: Failure to involve hard-to-reach populations in clinical research denies the potential benefits of research to the excluded groups, perpetuating health disparities. Employing community health workers (CHWs) may be an effective strategy to increase outreach and engagement of marginalized groups. CHWs are members of the target communities with a personal commitment to help their neighbors, and who serve as informants and communicators among their peers. CHWs may be particularly effective in addressing individual and cultural barriers to research participation. Because of their unique background and community-based roles, tailored training programs for CHWs are needed. The Recruitment, Retention, and Community Engagement Program at the UIC Center for Clinical and Translational Sciences seeks to train CHWs to be involved in the recruitment and enrollment of participants in clinical trials. We developed an 8-hour training that covers basic research methods (e.g., randomized clinical trials, longitudinal studies); research activities (e.g., surveys, interviews); and research ethics. The training focuses on the development of communication skills necessary for ethical recruitment and informed consent, providing strategies for addressing mistrust, fear and misunderstanding around the research process. Aim 1: To evaluate the feasibility of the CHW training by assessing. Aim 1.1: Recruitment of participants; Aim 1.2: Completion of training session (8 hr). Aim 2: To evaluate acceptability of training by assessing. Aim 2.1: Satisfaction with training; Aim 2.2: Cultural competence of training content; Aim 2.3: Participant self-efficacy in reproducing information. Aim 3: To collect performance measures by assessing. Aim 3.1: Knowledge gain and retention; Aim 3.2: Self-efficacy in identifying and addressing negative beliefs about research; Aim 3.3: Participants’ readiness to refer and/or recommend participation in clinical trials. METHODS/STUDY POPULATION: Methods: This is a pilot study with a single-group repeated-measures design with assessments at baseline, 1 week post-test, and 3- and 6-month follow-ups. We aim to recruit 25 CHWs working with organizations serving the needs of ethnic minorities in Chicago. We will evaluate feasibility (recruitment, completion of training and assessments) and acceptability of the training (satisfaction with training, cultural appropriateness of content and delivery, participant self-efficacy in reproducing information). Performance measures assessed through self-administered surveys at baseline, 1 week post-training, 3 months, and 6 months will include knowledge, attitudes toward research, and self-efficacy in identifying and addressing barriers to participation. Readiness to recruit and obtain informed consent will be assessed during an observed simulation activity with a standardized participant. Data analysis: Demographic data will be collected, and descriptive and inferential analyses will be conducted. Pretest and post-test questionnaire data will be compared using *t*-tests. In the informed consent simulation, individuals will be scored on whether they adequately addressed required elements of the informed consent process. Data gathered from the informed consent simulation will also be used for program evaluation and formative purposes; feedback on strengths and areas for improvement will be provided to participants. RESULTS/ANTICIPATED RESULTS: Expected results: It will be feasible to implement the training of CHWs, reaching the expected goal of 25 participants, with at least 70% of them completing the 8-hour training. We expect to collect data demonstrating acceptability of the training with a score of “good” or “excellent” by 70% of participants. At least 70% will rate the training as “culturally acceptable” or better, and will show improved self-efficacy in the delivery of information from pretest to post-test by at least 30%. Performance measures will demonstrate improvements in research knowledge by 30% from pretest to post-test; increased self-efficacy in identifying and addressing negative beliefs about research process, by at least 30%. A minimum of 70% of participants will demonstrate readiness to refer and/or recommend participation in clinical trials by scoring at or above 70% in evaluation of performance with standardized participants. Evaluation of knowledge retention at 3 and 6 months post-training will not take place before the Translational Science Conference in March 2018. DISCUSSION/SIGNIFICANCE OF IMPACT: Discussion/Impact. The outcomes of this evaluation may advance our knowledge of community obstacles to participation in research, and shed light on successful strategies to address them. Information obtained will be used to address limitations of the training. Even though the sample is small we expect to identify trends in quantitative measures that will support an application for funding for a larger randomized study. Once we have developed an effective training model, we expect to disseminate it to other CTSAs for broad implementation.

